# Global analysis of ZNF217 chromatin occupancy in the breast cancer cell genome reveals an association with ERalpha

**DOI:** 10.1186/1471-2164-15-520

**Published:** 2014-06-24

**Authors:** Seth Frietze, Henriette O’Geen, Laurie E Littlepage, Catalina Simion, Colleen A Sweeney, Peggy J Farnham, Sheryl R Krig

**Affiliations:** School of Biological Sciences, University of Northern Colorado, Greeley, CO 80639 USA; Genome Center, University of California, Davis, Davis, CA 95616 USA; Harper Cancer Research Institute, Department of Chemistry and Biochemistry, University of Notre Dame, South Bend, IN 46617 USA; Department of Biochemistry and Molecular Medicine, UC Davis School of Medicine, University of California, Sacramento, CA 95817 USA; Norris Comprehensive Cancer Center, Dept. of Biochemistry & Molecular Biology, University of Southern California, Los Angeles, CA 90089 USA

**Keywords:** Breast cancer, ZNF217, ERalpha, GATA3, FOXA1, ChIP-seq, RNA-seq, Endocrine resistance

## Abstract

**Background:**

The ZNF217 gene, encoding a C_2_H_2_ zinc finger protein, is located at 20q13 and found amplified and overexpressed in greater than 20% of breast tumors. Current studies indicate ZNF217 drives tumorigenesis, yet the regulatory mechanisms of ZNF217 are largely unknown. Because ZNF217 associates with chromatin modifying enzymes, we postulate that ZNF217 functions to regulate specific gene signaling networks. Here, we present a large-scale functional genomic analysis of ZNF217, which provides insights into the regulatory role of ZNF217 in MCF7 breast cancer cells.

**Results:**

ChIP-seq analysis reveals that the majority of ZNF217 binding sites are located at distal regulatory regions associated with the chromatin marks H3K27ac and H3K4me1. Analysis of ChIP-seq transcription factor binding sites shows clustering of ZNF217 with FOXA1, GATA3 and ERalpha binding sites, supported by the enrichment of corresponding motifs for the ERalpha-associated cis-regulatory sequences. ERalpha expression highly correlates with ZNF217 in lysates from breast tumors (n = 15), and ERalpha co-precipitates ZNF217 and its binding partner CtBP2 from nuclear extracts. Transcriptome profiling following ZNF217 depletion identifies differentially expressed genes co-bound by ZNF217 and ERalpha; gene ontology suggests a role for ZNF217-ERalpha in expression programs associated with ER^+^ breast cancer studies found in the Molecular Signature Database. Data-mining of expression data from breast cancer patients correlates ZNF217 with reduced overall survival.

**Conclusions:**

Our genome-wide ZNF217 data suggests a functional role for ZNF217 at ERalpha target genes. Future studies will investigate whether ZNF217 expression contributes to aberrant ERalpha regulatory events in ER^+^ breast cancer and hormone resistance.

**Electronic supplementary material:**

The online version of this article (doi:10.1186/1471-2164-15-520) contains supplementary material, which is available to authorized users.

## Background

Recurrent and common genomic amplifications have led to the discovery of important oncogenes and cancer therapeutic targets. The 20q13 amplification including the *ZNF217* gene coding for a transcription factor is found in ~ 20-30% of breast cancers and is associated with aggressive tumor behavior, shorter disease-free survival, chemoresistance, and poor prognosis [1, 2]. A recent report shows that ZNF217 overexpression accelerates aberrant cell differentiation through signaling events leading to increased self-renewal capacity, a mesenchymal phenotype, motility, chemoresistance and metastasis in mammary mouse models [3]. Earlier work using ChIP-chip tiling arrays for the 5 kb DNA region surrounding the transcriptional start site (TSS) identified ZNF217 regulatory gene targets in the embryonal carcinoma cell line, Ntera2, and the breast cancer cell line MCF7 [4]. This work supported a developmental role for ZNF217 as a regulatory factor at differentiation-specific genes. Findings from this work led to the discovery that ZNF217 directly activates *ERBB3* and downstream signaling events through PI3K and MAPK pathways [3, 5, 6]. Despite increasing knowledge of ZNF217-induced phenotypes and regulation of pathways promoting tumorigenesis, there is a lack of understanding of the downstream ZNF217-induced effectors driving these cell pathways.

ZNF217 encodes a transcription factor with eight C_2_H_2_ zinc finger motifs and a proline-rich transactivation domain at the C-terminus [7]. ZNF217 has been reported to physically interact with CtBP1/2 [7], an adaptor protein found in multiple regulatory complexes at both activated and repressed gene targets [5, 7, 8]. ChIP-chip studies indicate CtBP and ZNF217 are co-bound at the majority of ZNF217 DNA binding sites [4]. ZNF217 biochemically purifies with histone deacetylases HDAC1/2 [9, 10], histone demethylases LSD1 [10, 11] and Jarid1b/Plu-1 [11], and histone methyltranferases G9a and EZH2 [11], suggesting a range of regulatory functions in histone modifying complexes. Based on our current knowledge of ZNF217 and its association with DNA regulatory proteins, it has been hypothesized that ZNF217 functions as an organizer of histone chromatin modifiers [11]. The cooperating transcriptional mechanisms used by ZNF217, and its association with specific regulatory elements remains unexplored.

An important step in understanding the molecular role of ZNF217 in breast cancer is to gain a more complete understanding of the mechanisms of genome-wide gene regulation by ZNF217 in breast cancer cells, including the involvement of cooperating transcriptional partners. In the current study we employed an integrative genomics approach to uncover the mechanisms of ZNF217 target gene regulation in breast cancer cells. Using a combination of RNA- and ChIP-seq techniques in MCF7 breast cancer cells, we focused on identifying genes that were regulated by ZNF217 DNA binding. Our findings suggest a functional association for ZNF217 with estrogen receptor alpha (ERα) at co-bound ERα gene targets. This work supports further exploration into the connection between ZNF217 expression levels in breast tumors with clinical outcome and, importantly, whether ZNF217 plays a transcriptional role in aberrant ERα signaling, contributing to breast cancer and therapy resistance.

## Results

### ZNF217 genome-wide binding patterns

**Figure 1:**
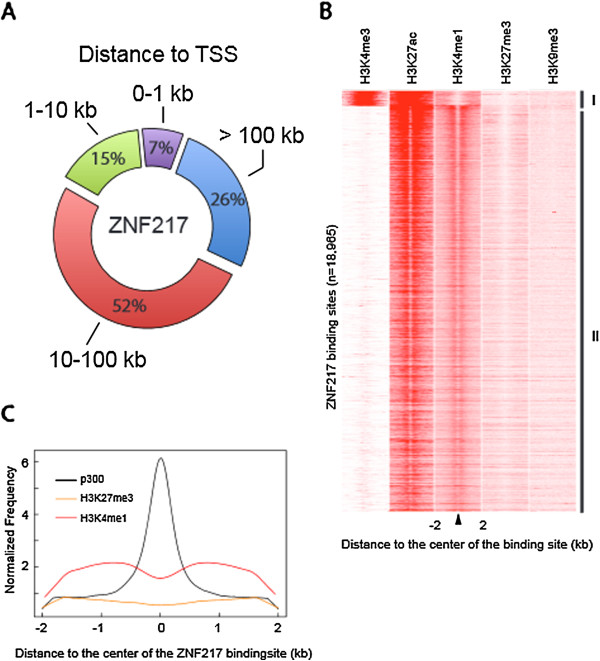
**Genome-wide analysis of ZNF217chromatin occupancy. (A)** Distribution of ZNF217 binding sites relative to the nearest annotated TSS. **(B)** Heatmaps depict H3K4me3, H3K27ac, H3K27me3, and H3K9me3 ChIP-Seq signals at regions spanning ZNF217 binding sites (±2 kb) in MCF7 cells organized according to their chromatin status: cluster I and cluster II. Active K4me3-only (cluster I) sites are predominately promoters, while K4me1 and K27ac sites (cluster II) are primarily enriched for distal regions. **(C)** Average p300 (black), H3K4me1 (red), and H3K27me3 (orange) ChIP-seq enrichment profiles around central position of ZNF217 binding sites.

To elucidate the regulatory function of ZNF217 in breast cancer, we performed genome-wide chromatin mapping of ZNF217 by ChIP-seq using the ER+ HER2- MCF7 cell line. Accordingly, we sequenced two independent ChIP-seq replicates and determined a set of enriched ZNF217 binding sites, using the Sole-Search peak calling tool [12], by taking the shared ZNF217 binding sites (False Discovery Rate (FDR) < 0.0001) from both replicates (18, 965 binding sites; Additional file 1). These datasets agree with the ENCODE guidelines for transcription ChIP-seq overlap rules [13]. To determine the Refseq genes nearest to each ZNF217 binding site, we used the Sole-Search location analysis tool. This analysis identified ZNF217 ChIP-seq targets at a total of 6,965 target genes with multiple binding events occurring at 3,619 genes (see Additional file 2). Our earlier work from MCF7 ChIP-chip promoter arrays identified ZNF217 binding at the proximal promoter regions of 5,061 genes [4]; 45% of these genes are present in the current ZNF217 ChIP-seq dataset. Analysis of distances between ZNF217-associated regions and the nearest annotated TSSs revealed that ZNF217 binds predominantly distal to promoter regions; less than 10% of the sites are located within 1 kb of annotated TSSs. In contrast, 15%, 52%, and 26% of the sites are located within 1–10 kb, 10–100 kb, and > 100 kb from a TSS, respectively (Figure 1A). Interestingly, location analysis shows 6,348 binding sites (33%) are located within genes (introns and exons), with the majority (92%) within introns. A panel of ZNF217-bound sites located at distal regions, including intronic sites, was confirmed by ChIP qPCR (see Additional file 3). ZNF217 binding at the proximal region of *TFF1* and the previously identified ZNF217 regulatory target, *ERBB3* [5], are also shown.

To further explore ZNF217 binding with respect to specific regulatory elements (such as enhancers or silencers), we analyzed the ChIP-seq read density relative to the total number of ZNF217 binding sites in MCF7 using ChIP-seq datasets for a number of activating histone marks including mono- and trimethylation of histone H3 Lys4, acetyl histone H3 Lys27 (H3K4me1, H3K4me3, H3K27ac, respectively), as well as the repressive histone marks trimethylation of histone H3 Lys9 and trimethylation of histone H3 at Lys27 (H3K9me3 and H3K27me3, respectively). The heatmap in Figure 1B shows a strong enrichment for H3K27ac and H3K4me1 relative to the center of the majority of ZNF217 sites (cluster II). A small proportion of sites are also enriched for H3K4me3 and H3K4me1 (cluster I). Location analysis on these ZNF217 binding sites indicate that the cluster II binding sites are largely distal regions, whereas the H3K4me3 enriched cluster I sites are largely promoter proximal (see Additional file 4). These results show that the majority of ZNF217 binding sites are marked by active chromatin marks and that the predominant class of binding events is located at distal regulatory elements associated with H3K4me1 and H3K27ac. Recent reports demonstrated that active enhancer elements are marked by the histone acetyltransferase p300 and by enrichment of H3K4me1 at flanking nucleosomes [14, 15]. Indeed, using p300 ChIP-seq data we observe p300 enrichment at ZNF217 peaks and H3K4me1 flanked ZNF217-bound regions, whereas H3K27me3 showed no substantial enrichment (Figure 1C).

To glean insight into the function of ZNF217 binding, we performed functional annotation of the top 5,000 ZNF217-bound regions with GREAT, a software tool designed to assign biological function to noncoding genomic regions [16]. This analysis showed an association with ontological categories including mesoderm development, mammary gland development and gastrulation. The Pathway Commons category terms aligned with ERα signaling and FOXA1 transcription networks. ZNF217 binding sites associated with multiple genes in TGFβ, WNT, and ER signaling pathways (gene associations are shown in Additional file 5).

### ZNF217 binding sites are occupied by multiple transcription factors

**Figure 2:**
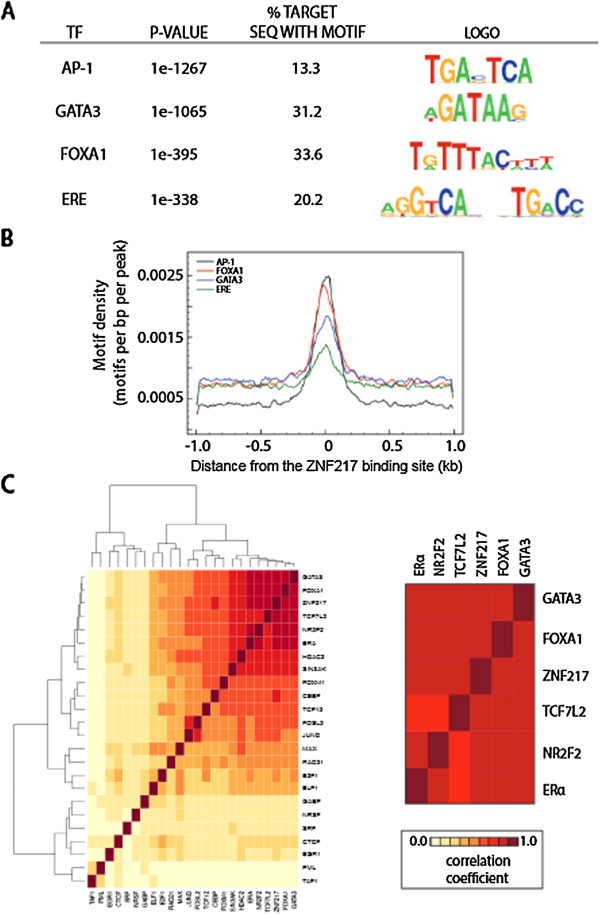
**ZNF217 binding sites cluster with transcription factor binding sites in MCF7 cells. (A)** Top TF motifs enriched in ZNF217 binding sites. **(B)** Distribution of identified motifs relative to the center of ZNF217 binding sites. Shown below are the motif logos for each TF. **(C)** ZNF217 shows co-binding with GATA3, FOXA1, TCF7L2, NR2F2 and ERα. Colors in the heat map reflect the colocalization frequency of each transcription factor (the descending frequency of localization ranges from red to yellow to white).

Another property of enhancer regions is their relative abundance of binding sites for multiple transcription factors [15, 17]. To gain insight into the function of ZNF217 at distal regulatory regions in relation to other transcriptional partners, we identified the predominant motifs for known transcription factors near ZNF217 binding sites. We retrieved the 200 bp DNA sequence for the ZNF217-bound regions and identified the most significant motif logos using HOMER [17] (Figure 2A). Our search identified enriched cis-regulatory sequences for FOXA1 in 31%, GATA3 in 34%, and EREs at 20% of the ZNF217 binding sites (Figure 2A) of which the density of each motif is enriched ± 200 bp relative to the center of the ZNF217 binding peak (Figure 2B). Next, we searched for *de novo* motifs over-represented within ZNF217-bound regions. We did not find enrichment of the ZNF217 consensus sequences reported in our earlier work [4], or the work of others [8]. We did however identify a unique, non-repetitive sequence at ZNF217 sites: 5’-TGA(G/C)TCA(T/C)-3’ (or its reverse complement 5’-(A/G)TGA(C/G)TCA-3’). This *de novo* ZNF217 consensus site indicates enrichment of a sequence similar to the AP-1 motif (see Additional file 6). AP-1 motifs are very often found near binding sites for many types of TFs, including ERα, and AP-1 has been considered by some as a “general” enhancer binding factor [18]. AP-1 is a transcription factor that interacts with ERα and plays a role in the recruitment of ERα upon ligand binding to specific regulatory elements within the genome [18–21]. To further investigate the transcriptional partners of ZNF217, we performed unsupervised clustering analysis of all ENCODE ChIP-seq transcription factor binding sites found in MCF7 cells. We found a group of five transcription factors with binding sites that cluster with ZNF217 binding sites, including TCF7L2, NR2F2, ERα, GATA3, and FOXA1 (Figure 2C). This occupancy and motif analysis highlights a significant interaction between ZNF217 and alternative breast cancer transcription regulators.

**Figure 3:**
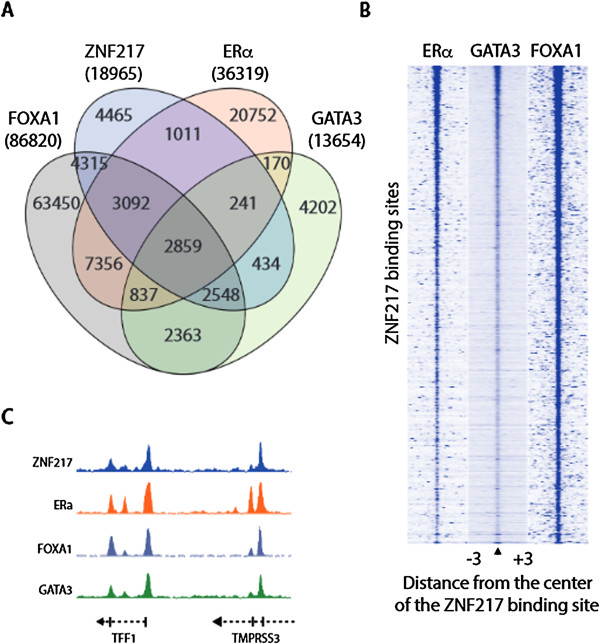
**ZNF217 binding correlates with ERα, GATA3 and FOXA1. (A)** A four-way comparison of binding sites shared with ZNF217 in MCF7 cells. Listed below the factor is the total number of binding sites for that dataset. **(B)** Heatmap depicting ERα, GATA3 and FOXA1 ChIP-seq signal density spanning ZNF217 binding sites (±2 kb) in MCF7 cells. **(C)** IGB snapshot of ZNF217, ERα, GATA3 and FOXA1 occupancy at the *TFF1* gene.

Several proteins serve as pioneer factors contributing to ERα action in breast cancer, including FOXA1 and GATA3 (reviewed in [22]). We therefore assessed the four-way overlap between these factors. Binding site overlap analysis indicate a large fraction of the total ZNF217 binding sites are shared by ERα, GATA3, and FOXA1 in asynchronous MCF7 cells (Figure 3A and C). In addition, this analysis reveals distinct combinations of binding sites shared between these factors. We found that while 41% of ZNF217 sites overlap with ERα (see Additional file 7), there are other unique associations of ZNF217 with GATA3 and FOXA1 independent of ERα in MCF7 cells (Figure 3A). The individual sets of genes bound by ZNF217 and each factor or groups of factors are listed in Additional file 8. Heatmap clustering of the read density from independent ERα, FOXA1, and GATA3 ChIP-seq experiments supports the association of these transcriptional regulators, where an enriched signal is prevalent surrounding the center of ZNF217 binding sites (Figure 3B). One example of overlap between ZNF217 with ERα, FOXA1, and GATA3 co-bound regions is shown at the *TFF1* gene, a well-known ER gene target [23] (Figure 3C).

### Depletion of ZNF217 identifies a gene expression signature associated with ERα gene targets

**Figure 4:**
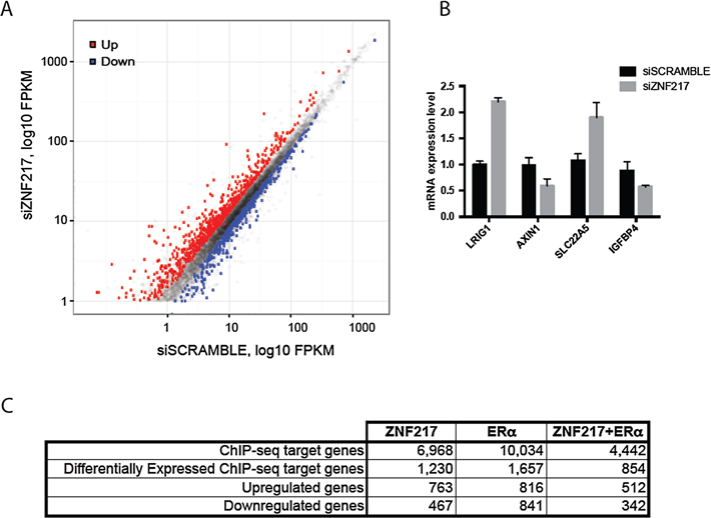
**Analysis of gene expression changes upon ZNF217 knockdown in MCF7 cells. (A)** A scatterplot of expression data from RNA-seq experiments. Each point corresponds to one NCBI Reference Sequence (RefSeq) transcript with fragments per kilobase of exon model per million mapped reads (FPKM) values for control and ZNF217 knockdown samples shown on an exponential scale. Significantly affected transcripts (FDR < 0.05 with a 1.25-fold change cuttoff) are depicted (red upregulated and blue downregulated upon ZNF217 knockdown). The dashed line represents no change in gene expression between the two samples. **(B)** The triplicate biological (sequenced) RNA samples from siScrambled- or siZNF217- treated MCF7 cells were analyzed by quantitative RT-PCR. ABI expression assay Taqman probes to measure transcript levels of six genes are indicated. Each sample was assayed in triplicate and the cT values were normalized to GAPDH. Average relative transcript level was graphed using BioRad CFX software. *Columns*: transcript levels, *error bars*: standard error of the mean. **(C)** Summary of differentially expressed genes, downregulated or upregulated by depletion of ZNF217 and bound by ZNF217, ERα, or both ZNF217 and ERα.

To gain further insight into the regulation of gene expression by ZNF217, we employed an RNA-seq approach following ZNF217 silencing to characterize the direct and indirect targets of ZNF217 in MCF7 breast cancer cells. We treated cells with ZNF217-targeting short interfering RNA (siRNA) leading to ZNF217 mRNA and protein depletion (see Additional file 9) and performed gene expression profiling by RNA-seq. We identified 3,402 differentially expressed genes; 1,615 genes were up-regulated and 1,787 were down-regulated genes in the ZNF217 knockdown sample, as compared to the control (siScramble) siRNA-treated sample (at 1.25 -fold change cutoff and criteria representing statistical significance, false discovery rate (FDR) < 0.0001; genes listed in Additional file 10; Figure 4A). RNA-seq results were confirmed by RT-qPCR on a small panel of genes (Figure 4B). To determine if the genes that changed with ZNF217 silencing are also bound by ZNF217, we integrated the ChIP-seq and RNA-seq datasets and compared the expression of genes with a ZNF217 binding site. Among the 3,402 differentially expressed genes, following ZNF217 silencing, 1,230 (36%) of the ZNF217-bound genes change with ZNF217 knockdown. In addition, we find that 62% and 38% of the 1,230 differentially expressed genes with a ZNF217 binding site show increased and decreased expression, respectively (Figure 4C). We also found that among a total 4,442 genes co-bound by ZNF217 and ERα, 854 genes change in expression with ZNF217 silencing of which 60% (512 genes) show increased expression levels (Figure 4C). Independent RT-qPCR experiments validate the increased levels of the known estrogen responsive ERα-target genes LRIG1 [24] and SLC22A5 [25] and the decreased levels of AXIN1 and IGFBP4 following ZNF217 depletion (Figure 4B and Additional file 11).

### The ZNF217 gene signature identifies gene set enrichments from ER^+^ breast cancer studies

**Figure 5:**
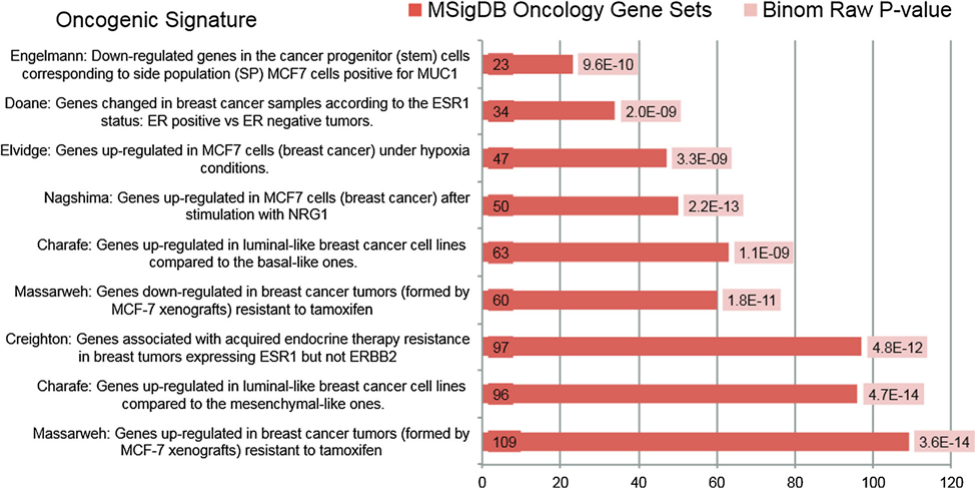
**Analysis of gene expression changes upon ZNF217 knockdown in MCF7 reveals deregulation of breast cancer gene signatures.** GREAT gene ontology on the 854 ZNF217-ERα co-bound gene list identifies overlap with several MSigDB Cancer Studies (p < 10^−8^, exact binomial test).

To understand the genes regulated by ZNF217 and the pathways associated with ERα function, we performed gene ontology analysis on the lists of differentially regulated genes following ZNF217 silencing with the GREAT classification gene ontology tool [16]. Interestingly, both the list of 1,230 ZNF217-bound differentially regulated genes and the list of 854 ZNF217-ERα co-bound differentially regulated genes show strong associations with genes associated with multiple cancer studies present in the Molecular Signatures Database (MSigDB) [26]. Genes in the ZNF217-ERα co-bound list correlate with genes identified from key cancer studies including: (a) Genes found up-regulated or down-regulated in breast cancer tumors (formed by MCF-7 xenografts) resistant to tamoxifen (Massarweh [27]), (b) Genes up-regulated in luminal-like breast cancer cell lines compared to basal or mesenchymal-like ones (Charafe [28]), (c) Genes associated with acquired endocrine therapy resistance in breast tumors expressing ESR1 but not ERBB2 (Creighton [29]), (d) Down-regulated genes in the cancer progenitor (stem) cells corresponding to side population (SP) MCF7 cells positive for MUC1 (Engelmann [30]), (e) Genes up-regulated in MCF-7 cells under hypoxia and (Elvidge [31]), (f) Genes that change according to the ESR1 status: ER positive vs ER negative tumor (Doane [32]), and (g) Genes up-regulated in MCF7 cells (breast cancer) after stimulation with NRG1 (Nagashima [33]). The enrichments of the ZNF217-ERα co-bound MSigDB categories are shown in Figure 5. A comparison of the complete list of cancer studies associated with ZNF217-bound and ZNF217-ERα co-bound genes are in Additional file 12.

We were particularly interested in the combined up- and down-regulated ZNF217 differentially expressed genes (169) associated with the Massarweh expression study [27]. Gene ontology for the 169 genes showed functional classification associating with developmental pathways; many of these genes are known estrogen-responsive ERα target genes (data not shown). The gene expression changes found in tamoxifen-resistant tumors (derived from MCF7 xenografts) included several genes either up or down-regulated by ZNF217 and co-bound by ZNF217- ERα including *CAV2, ELF5, ID2, TGFB2, LRIG1, FOXO3, CXCL12, and CD44*.

### ZNF217 and ERα precipitate in MCF7 nuclear extracts

Our genome-wide binding analysis indicates ZNF217 is associated with ERα and other regulatory factors at “active” DNA regulatory elements. It is well-known that nuclear receptors do not function in isolation but require co-factors to assist with DNA interaction; intensive work in the field of estrogen receptor biology has sought to identify critical factors that affect ERα-mediated transcription [34]. To identify potential unknown regulators of ERα, Jason Carroll’s group performed a novel approach termed RIME (Rapid Immunoprecipitation with ERα followed by Mass spectrometry of Endogenous proteins). This work identified an association between ERα and 108 proteins, including the association of ZNF217 and the binding partners, CtBP1/2, in three out of three replicate experiments [35]. To confirm this finding, we tested whether or not ZNF217 associates with ERα in MCF7 nuclear extracts by co-immunoprecipitation assays. Immunoblotting of lysates showed ERα precipitated both ZNF217 and the ZNF217-binding partner CtBP2 (see Additional file 13).

### ZNF217 is enriched in ER^+^ breast cancer

**Figure 6:**
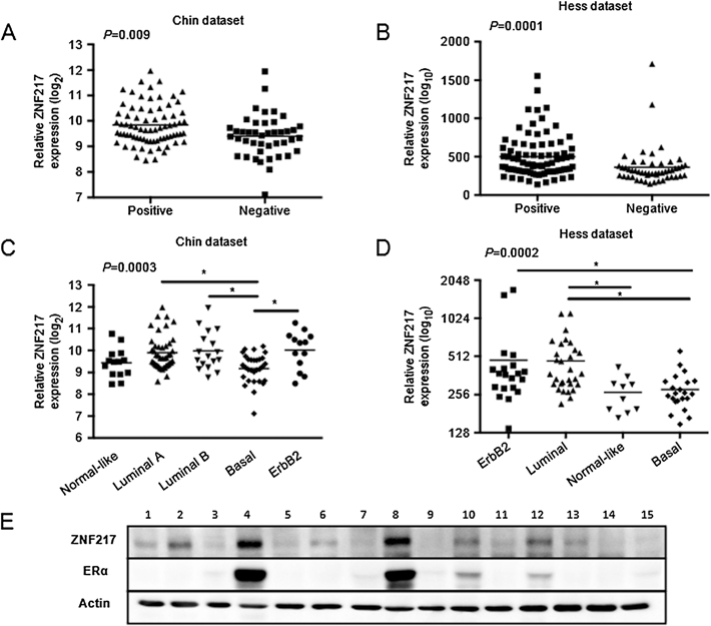
**High ZNF217 expression is enriched in ER + breast cancer. (A)** Estrogen receptor status from Chin *et al*. 2006 study [36]. ZNF217 expression levels in primary tumors that were ER positive (n = 75) or ER negative (n = 43) compared by Mann–Whitney t-test (p = 0.009). **(B)** Estrogen receptor status from Hess *et al*. 2006 study [37]. ZNF217 expression levels in primary tumors that were ER-positive (n = 82) or negative (n = 51) and compared by Mann–Whitney t-test (p ≤ 0.001). **(C)** ZNF217 expression levels in patients separated by gene expression molecular subtypes (p = 0.0003; ANOVA,Kruskal-Wallis) from Chin *et al*. 2006 [36] study. **(D)** ZNF217 expression levels in patients separated by gene expression molecular subtypes (p = 0.0002; ANOVA,Kruskal-Wallis) from Hess *et al*. 2006 [37] study. For both analyses, individual cohort combinations were compared by Dunn’s multiple comparison test, and those marked with * had p < 0.05. Each line marks the mean for the subtype. **(E)** Immunoblotting of 15 breast tumor lysates with antibodies to ZNF217, ER, or actin as a loading control. ZNF217 and ER protein densitometry was quantitated (r^2^ = 0.93).

If ZNF217 interacts with ERα and potentially contributes to the chromatin landscape associated with ERα binding, then we reasoned that ZNF217 expression might be higher in estrogen receptor positive (ER^+^) tumors. Using microarray expression studies of breast tumors across multiple patient cohorts, we found that ZNF217 expression levels are higher in ER^+^ tumors than in ER^-^ tumors (data-mining from the Chin 2006 [36] and the Hess 2006 [37] studies are shown in Figure 6A and B). We next compared ZNF217 expression levels across patient subtypes: Luminal A, Luminal B, ERBB2^+^, basal, and normal. Patient data from the Chin and Hess studies indicate ZNF217 expression is consistently highest in Luminal and lowest in basal subtype tumors (Figure 6C and D).

We previously found that high ZNF217 expression is a prognostic indicator in general as well as in ER^+^HER2^+^LN^−^ patients with breast cancer correlating with shorter overall, disease-specific, and relapse-free survival [3]. We next wanted to determine if ZNF217 overexpression had prognostic value exclusive of estrogen receptor status. We found that high ZNF217 expression consistently predicted poor prognosis across multiple tumor subtypes: Luminal, ER^−^, HER2^+^/ERBB2^+^, and basal cohorts ([3] and Additional file 14). Together, our data show that ZNF217 is prognostic of reduced survival in ER^+^, ER^−^, HER2^+^/ERBB2^+^, Luminal, and basal subtype patients by univariate analysis. Moreover, ZNF217 was a better predictor of survival than ERα status but not tumor size by multivariate analysis [3].

We also looked for a correlation between ZNF217 and ERα protein expression in breast tumor samples. We screened a small sample size of 15 tumor lysates and immunoblotted for ZNF217 and ERα. Densitometry indicated a high correlation for ZNF217 and ERα protein expression (r^2^ = 0.93) in the tumor samples (Figure 6E).

### ZNF217 expression is prognostic of reduced survival in patients with ER^+^ and Luminal A breast tumors

**Figure 7:**
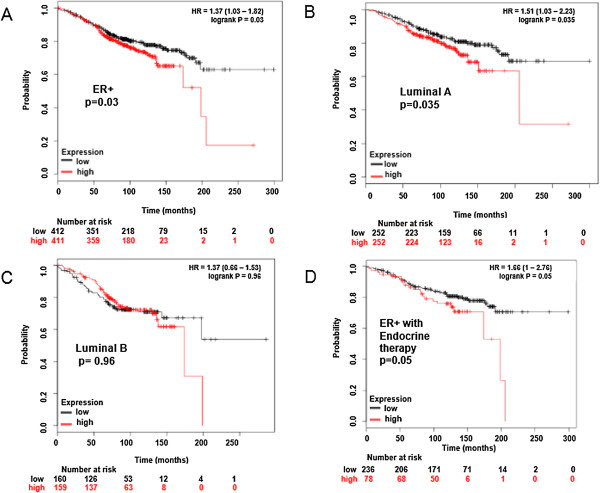
**ZNF217 expression is prognostic of reduced survival in patients with ER** ^**+**^ **and Luminal A breast tumors.** ZNF217 gene expression was analyzed using the Kaplan Meier-Plotter, an online survival analysis tool to analyze gene expression with breast cancer prognosis using microarray data from 1,809 patients [38]. High ZNF217 expression affects Overall Survival in Luminal A but not Luminal B ER^+^ breast cancer patients (any systemic anti-hormonal therapy treatment and/or chemotherapy). The prognostic value of high ZNF217 expression (median cutoff) was compared to the patient cohort with low ZNF217 expression in **(A)** All ER^+^ patients (n = 823), p = 0.03. **(B)** Luminal A (n = 504), p = 0.035 **(C)** Luminal B (n = 319), p = 0.96. **(D)** High ZNF217 correlates with worse survival in ER^+^ patients treated with any endocrine therapy (but not chemotherapy). The prognostic value of high ZNF217 (n = 78) expression (upper quartile) was compared to the patient cohort with low ZNF217 expression (n = 236), p = 0.01.

To further explore the effect of ZNF217 expression on patient survival we interrogated the Kaplan Meier-plotter, an online survival analysis tool, for overall survival in breast cancer patients to rapidly assess the effect of 22,277 genes on breast cancer prognosis using microarray data of 1,809 patients [38]. We compared all ER^+^ patients (all intrinsic subtypes with any therapy) and found that high ZNF217 expression was associated with lower overall survival (p = 0.03, Figure 7A). To determine whether ZNF217 was simply identifying the poorer prognosis Luminal B tumors from within the Luminal cohort, we asked whether ZNF217 expression had prognostic value in the ER^+^ Luminal A tumors or the ER^+^ Luminal B tumors (with any chemo or anti-hormone therapy). As shown in Figure 7B, high ZNF217 expression is associated with lower overall survival in the Luminal A tumors (p = 0.035). Interestingly, the data for Luminal B tumors expressing high ZNF217 did not reach statistical significance (Figure 7C). This finding suggests that ZNF217 provides additional prognostic information, beyond ERα status and Luminal subtype.

### ZNF217 expression is prognostic of reduced survival after hormone treatment of ER^+^ breast cancer patients

The high overlap between differentially regulated genes co-bound by ZNF217 and ERα and the poor prognosis of patients with breast tumors that overexpress ZNF217 suggested that ZNF217 may have a regulatory role in expression changes that occur in tamoxifen-resistant breast tumors. To determine if high ZNF217 expression correlates with reduced overall survival in the cohort of ER^+^ patients that received anti-hormone therapy *only* (without the confounding effects of chemotherapy), we explored n = 78 patients with high versus n = 236 low ZNF217 expression in the Kaplan Meier-plotter (Figure 7D). Patients with high ZNF217 expression (upper quartile) demonstrated worse outcome p = 0.05.

## Discussion

In this work we have mapped the chromatin landscape surrounding ZNF217 DNA binding sites and found significant overlap between ZNF217 binding and transcription factors associated with the ERα regulatory network. ZNF217 predominantly occupies distal regulatory regions marked by the active histone marks H3K27ac and H3K4me1 and, depending on the chromosomal context, may participate either in the repression or the activation of gene expression. A similar mode of action for ER-mediated gene regulation has been proposed where ERα associates with auxiliary TFs and co-regulators in a specific chromosomal context to control gene expression, although it is not well understood what specifies the context-dependent activity of this regulation. In support of a transcriptional role for ZNF217 in aberrant ERα signaling, we identified a ZNF217-ERα gene signature with ontological classification that aligns with multiple breast cancer studies (Figure 5). Data-mining of expression data from breast cancer patients also associates high ZNF217 expression with poor prognosis across multiple intrinsic subtypes and reduced response to hormone therapy (Figure 7).

The function of ER binding at chromatin regions and transcriptional regulation is strongly influenced by GATA3 and FOXA1 [39–42]. GATA3 appears to function as a critical regulator of transcription factor binding, chromatin structure, and long-range genomic communication [43]. FOXA1 is known as a pioneer factor, a special class of transcription factors that bind to condensed chromatin and facilitate the binding of additional transcription factors [22]. An elegant study by Ross-Innes *et al.*, mapped the genome-wide ER-binding events by ChIP-seq in primary breast cancers from patients with different clinical outcomes [44]. They found distinct ER binding co-factor combinations linked with the different clinical outcomes, and that FOXA1 appears to mediate the differential ER-binding profiles found in clinical subtypes. However it is still unknown what dictates the differential FOXA1 and subsequent ERα binding. At the genome level approximately a quarter of all ERα -binding events are co-occupied by GATA3 and FOXA1 [43]. Similarly, we found an enrichment of GATA3 and FOXA1 factors at ZNF217-bound regions (Figure 3A). Our results suggest ZNF217-bound regions may be co-occupied with ERα and FOXA1 at multiple ERα gene targets, including *TFF1, ERBB3, SLC22A5,* and *LRIG1* (Additional file 3: Figure S1B and Additional file 11: Figure S6). Knockdown of ZNF217 followed by expression profiling identified 3,402 differentially affected genes suggesting a functional regulatory role linked to multiple signaling pathways associated with breast cancer (Figure 5 and Additional file 12: Table S6). Future work will determine whether ZNF217 binding to ERα cis-regulatory sequences contributes an additional level of regulation to ERα-binding potential and ERα-signaling. Multiple regulatory scenarios are possible and include ZNF217 promotion of chromatin accessibility through recruitment of chromatin remodeling factors or regulation of three-dimensional chromatin to spatially connect distal enhancer regions with proximal promoters. It will be interesting to unravel the potential regulatory role for ZNF217 signaling and whether ZNF217 co-operates or competes with ERα regulatory factors such as GATA3 and FOXA1.

Interestingly, ZNF217 and GATA3-FOXA1 are associated with opposite tumor phenotypes. Well-differentiated tumors are generally less advanced and support a better prognosis than poorly differentiated tumors that are more aggressive and correlate with poorer prognosis. The regulation of cell differentiation during normal development involves transcription factors that are increasingly associated with tumorigenesis upon their aberrant re-activation and expression. Recently, GATA3 has been shown to play an important role at multiple stages of mammary gland development, including the formation of terminal end buds in luminal cell differentiation [45]. GATA3 is a strong predictor of tumor grade, ER status, well-differentiated tumors, and a marker of good prognosis. Loss of GATA3 coincides with loss of differentiation and tumor metastasis [45]. Similar to GATA3, FOXA1 plays a role in postnatal development of the mammary gland, perhaps due to its requirement for ERα chromatin binding and activity [46, 47]. Both GATA3 and FOXA1 expression are part of the Luminal A molecular subtype gene signature [48], associated with well-differentiated tumors and better prognosis. Tumors negative for GATA3 and FOXA1 expression associate with poorly differentiated tumors and malignant progression [45, 46].

In contrast to GATA3 and FOXA1, the ZNF217 gene is amplified at 20q13 in ~20% of breast tumors and is associated with aggressive breast disease [1, 3, 49]. Our recent studies show the overexpression of ZNF217 promotes cell plasticity particularly in the maintenance of the de-differentiated stem-like cell state, and accelerates tumor progression and metastasis [3]. Our findings that ZNF217 interacts physically with ERα and that ZNF217 occupies chromatin regions also occupied by ERα, GATA3, and FOXA1 will require further investigation to understand whether ZNF217 is one of the multiple factors that contribute to ERα chromatin binding and ERα gene regulation.

Data-mining of microarray expression data from primary breast tumors and the corresponding clinical data showed high ZNF217 expression correlates with shorter overall survival and relapse-free survival [3]. Our current studies demonstrate a high correlation between ZNF217 and ERα expression in breast cancer cell lines and breast tumor tissue, yet the precise role of ZNF217 in ER^+^ breast cancer is not fully understood. Studies conducted over 10 years ago using microarray technology revealed *tumor heterogeneity* at the gene expression level and led to the identification of breast cancer subtypes [48, 50, 51]. The ER^+^ subtypes, referred to as Luminal A and Luminal B, have different gene-expression profiles, prognosis and treatment responses [48, 50–52]. When compared to the Luminal A subtype, Luminal B tumors often have lower expression levels of ERα or ER-regulated genes, higher tumor grade, activation of growth factor receptor signaling pathways and reduced sensitivity to chemotherapy than Luminal A tumors [53]. Despite the divisions between Luminal A and B subtypes, *the heterogeneity found in* ER^+/−^*and luminal tumors suggests further stratification is necessary to enable more accurate prognosis and treatment plan for individual patients* [53]. Our findings suggest that ZNF217 overexpression in ER^+^, Luminal A, or other subtypes of breast cancer will identify a subset of patients with worse prognosis who will benefit from more aggressive or alternative therapy. The identification of the ZNF217-ER gene signature aligning with multiple breast cancer studies further supports a transcriptional role for ZNF217 in aberrant ERα signaling (Figure 5). Future investigations will explore the connection between ZNF217 expression levels in breast tumors with clinical outcome and, importantly, whether ZNF217 plays a transcriptional role in aberrant ERα signaling, contributing to breast cancer and therapy resistance.

## Conclusions

In this genome-wide study we show the distribution of ZNF217 binding events coincide with GATA3, FOXA1, and ERα binding. Motif analysis and co-immunoprecipitation assays implicate ZNF217 in a co-regulatory role with ERα at co-bound regions. The understanding of the chromatin landscape at ZNF217-bound regions is beginning to provide insight into a relationship between ZNF217 and ERα to modulate enhancer accessibility, ERα-binding potential, and gene regulation. Additional studies are underway to explore the requirement of ZNF217 in ERα gene regulation at co-bound sites prior to estrogen stimulation. Our findings contribute to the genome-wide potential of ZNF217 in the hierarchy of ERα-mediated regulation by multiple transcription factors and provide a possible mechanism by which ZNF217 contributes to poor prognosis in breast cancer patients.

## Methods

### Cell culture

The MCF7 breast cancer cell line was obtained from American Type Culture Collection and cultured in Dulbecco’s Modified Eagle’s Medium supplemented with 10% fetal bovine serum, 2 mM L-glutamine and 1% penicillin/streptomycin.

### Primary human tissue specimens

Frozen human breast tumor specimens were provided by the UCD Cancer Center Specimen Repository and the NCI Cooperative Human Tissue Network. All samples were de-identified, and the study was approved by the Institutional Review Board of the UCD School of Medicine. Frozen tissues were homogenized in ice-cold T-PER Tissue Protein Extraction Reagent (Pierce) with protease and phosphatase inhibitors, and then centrifuged to remove insoluble debris.

### siRNA-mediated silencing

MCF7 cells were reverse-transfected at a density of 5 × 10^5^ per well in 6-well culture plates. Transfection was performed using 100 nM siRNA targeting ZNF217 (OnTarget plus SMARTpool, Dharmacon, cat # L-004987-01) or non-targeting pool (OnTarget plus non-targeting pool, Dharmacon, cat # D-001810-10) using RNAiMax (Invitrogen) per the manufacturer’s instructions. RNA was collected at 48 h post-transfection.

### RNA isolation and real-time qPCR

RNA samples for RNA-seq or cDNA synthesis were purified using a commercial kit (Qiagen; RNAeasy Kit). RNA (2.5ug) was converted to cDNA using the High Capacity cDNA Reverse Transcription Synthesis Kit (Applied Biosystems). Analyses were performed using TaqMan Gene Expression Assays (Applied Biosystems; listed in Additional file 15: Supplemental Methods) on the BioRad CFX 9600 thermocycler.

### Co-immunoprecipitation assays

A total of 1x10^7^ MCF7 cells were grown in complete medium and harvested after washing with PBS. The cellular nuclear extracts were isolated with a Nuclear Extraction Kit and provided protocol (Affymetrix cat #AY2002). Immunoprecipitation was performed with 5 ug of antibody or negative IgG control from rabbit on pre-cleared nuclear extracts. After overnight incubation, protein G agarose beads were added for 2 h. The beads were washed in co-IP buffer (20 mM Tris ph 7.5,150 mM NaCL, 1% NP40,10% glycerol,1 mM MgCl_2_), boiled 5 min at 95°C and run on an 8% acrylamide gel. Antibodies: ZNF217 column-purified rabbit polyclonal [4], ERα (Santa Cruz Biotechnology; cat #scbt-543), CtBP2 (BD Transduction cat #612044), and IgG from rabbit serum (Sigma cat #15006).

### ChIP-seq assays

Chromatin immunoprecipitation (ChIP) was performed following the method previously described [54]. Briefly, crosslinked chromatin was collected from MCF7 cells asynchronously grown in full media at 80% confluence. ZNF217 antibody (previously described [4]), H3K4me3, H3K9me3, or H3K27me3 antibodies were incubated with sonicated chromatin overnight. Libraries were constructed and analyzed using an Illumina Hiseq2000. ChIP-seq experiments were performed in duplicate using MCF7 cells grown on different days using independently performed ChIP assays. Replicate input chromatin samples were also included as a control. Additional ChIP assays were performed for ERα (Santa Cruz Biotechnology; cat #scbt8002) and FOXA1 (Abcam #23738). ChIP-qPCR was performed using iQ SYBR Green Supermix (BioRad). Primers used to validate TF binding are listed in Additional file 15: Supplementary Methods.

### ChIP-seq data processing

All ChIP-seq datasets were processed by first aligning sequence reads to the hg19 genome using Bowtie2 [55]. We obtained the raw read data files for the ChIP-seq experiments corresponding to p300, ERα, FOXA1 and GATA3 from asynchronously grown MCF7 cells from previously published studies [41, 56, 57]. H3K27ac and H3K4me1 ChIP-seq data generated in asynchronously grown MCF7 cells was obtained from [57]. All transcription factor and histone modification peaks were called using Sole-Search software using sequenced MCF7 input as a control [12, 58]. For ZNF217, we used the ENCODE overlap rules to evaluate the reproducibility of the two biological replicates for each factor or histone modification and cell-type combination [13]. For this, we first truncated the peak lists of the two replicates for a given factor/cell-type combination so that both the A and B replicate peak list were the same length. Then, we overlapped the top 40% of the replicate A peak list with the entire replicate B peak list (and vice versa). ENCODE standards state that approximately 80% of the top 40% set should be contained in the larger set. After determining that replicate datasets met this standard, we merged the two replicates and called peaks on the merged dataset.

### TF Co-association

Co-occurance analysis to study the overlap of ZNF217 with other transcription factor binding sites was performed using the Aggregation and Correlation Tool (ACT Tool) [59]. The signal files corresponding all MCF7 transcription factor binding regions were obtained from the ENCODE project (https://genome.ucsc.edu/ENCODE/downloads.html). Using these signal tracks and peak lists for transcription factor we used the ACT tool to perform the signal aggregation over each peak list. The resulting matrix was clustered using Cluster3.0 [60] using a Pearson Correlation distance metric and was visualized in R.

### RNA-seq

RNA samples from triplicate biological replicates of MCF7 cells treated with scrambled siRNA or siZNF217 were extracted using the RNAeasy Kit (Qiagen). 2 ug of RNA samples were processed with the TruSeq RNA Sample Prep Kit (Illumina) to make RNA libraries following the Low-Throughput protocol. Libraries were prepared and sequenced using the HiSeq2500 platform at the UC Davis DNA Technologies Core Facility (http://dnatech.genomecenter.ucdavis.edu).

### RNA-seq data processing

RNA-seq data was processed using TopHat and Cufflinks [61, 62] as described [63].

### Additional supporting data

All data are publicly available via the UCSC Genome ENCODE Browser and/or has been submitted to the Gene Expression Omnibus. The NCBI accession number for the RNAseq data is GSE58326. http://www.ncbi.nlm.nih.gov/geo/query/acc.cgi?acc=GSE58326. The NCBI accession number for the ZNF217 ChIPseq data is GSM935563.

## Electronic supplementary material

Additional file 1: Table S1: ZNF217 binding sites from merged ChIP-seq datasets. ZNF217 peaks were called using Sole-Search peak calling software [12, 58] on replicate datasets (see Methods for details). A total of 18,965 ZNF217 binding events are listed. (XLS 2 MB)

Additional file 2: Table S2: ZNF217-associated genes (6,965) from ChIP-seq binding sites. (XLS 432 KB)

Additional file 3: Figure S1: ChIP-qPCR confirming TF binding sites identified by ChIP-seq. (A) Panel of ZNF217-bound distal sites confirmed by ChIP-qPCR. (B) Relative TF ChIP enrichment at the ERα target genes *ERBB3*, *TFF1*, and *LRIG1*. ChIP assays were performed on two independent biological replicates using the following antibodies ZNF217, ERα and FOXA1. IgG was used as a control. Relative DNA enrichment was calculated relative to DNA input and using the non-target ZNF10 locus as a negative control. (PDF 337 KB)

Additional file 4: Figure S2: Location Analysis of ZNF217 epigenomic clusters. Location analysis of the cluster I region and cluster II region from Figure 1B. The fraction of the ZNF217 binding sites found in cluster I (top chart) or cluster II (below chart) relative to Refseq genes is shown. (PDF 365 KB)

Additional file 5: Table S3: GREAT annotation of top 5 k ZNF217-associated genes. Table showing GO Biological Process category from gene analysis using the GREAT web tool. (XLSX 22 KB)

Additional file 6: Figure S3: *de novo* ZNF217 motif. Identification of *de novo* motif for ZNF217 binding sites in MCF7 cells. HOMER *de novo* motif analysis using the central most 100 base pairs of all 18,965 ChIP-seq binding regions reveal the indicated motif (shown in both forward and reverse complement). (PDF 389 KB)

Additional file 7: Figure S4: Venn diagram for ZNF217 and ER binding overlap. Overlap analysis of ZNF217 and ERα ChIP-seq binding sites in MCF7 cells. The Venn diagram illustrates the total number of genomic regions shared between these two factors (overlapping by at least 1 base pair). The total number of identified sites is indicated below each factor’s name. (PDF 355 KB)

Additional file 8: Table S4: 4-way Venn Diagram Gene Overlap for multiple TF binding. sites. Excel file contains multiple worksheets listing the ZNF217, ERα, FOXA1 and GATA3-associated genes and co-bound genes for TF combinations. (XLSX 13 MB)

Additional file 9: Figure S5: Validation of ZNF217 knockdown for RNA-sequencing. MCF7 cells were reverse transfected with scrambled or ZNF217 siRNA for 48 h. Cells were collected from each well and split into two samples for RNA isolation and protein lysate. (A) Protein lysates from triplicate samples were immunoblotted for ZNF217 or actin as a loading control. Chemoluminescence was analyzed on an Alpha-Innotech Imaging documentation system. (B) RNA samples were converted to cDNA and quantitative RT-PCR performed using ABI expression assay Taqman probes for *ZNF217* and *GAPDH*. Each sample was assayed in triplicate and the cT values were normalized to GAPDH. Average relative transcript level was graphed using BioRad CFX software. *Columns*: transcript levels, *error bars*: standard error of the mean. (PDF 651 KB)

Additional file 10: Table S5: Differentially expressed genes from RNA-seq. List of the 3,402 differentially expressed genes; 1,615 genes were up-regulated and 1,787 were down-regulated genes in the ZNF217 knockdown sample, as compared to the control siRNA-treated sample (at 1.25 -fold change cutoff and criteria representing statistical significance, false discovery rate (FDR) < 0.0001. (XLS 992 KB)

Additional file 11: Figure S6: Integrated Genome Browser snapshots of 4 genes bound by ZNF217, ERα, FOXA1 and GATA3. Co-bound regions are shown for *LRIG1*, *AXIN1*, *SLC22A5*, and *IGFBP4*. (PDF 489 KB)

Additional file 12: Table S6: MSigDB cancer studies for 1,230 and 854 gene lists. Worksheets with complete list of all MSigDB cancer studies resulting from GREAT analysis for ZNF217-bound genes versus ZNF217-ER co-bound genes. (XLSX 17 KB)

Additional file 13: Figure S7: ERα co-immunoprecipitation with ZNF217 and CtBP2. Co-immunoprecipitation of endogenous ZNF217, ERα and CtBP2 from MCF7 nuclear extracts. Immunoprecipitation experiments with ERα antibody to co-IP ZNF217 were blotted with corresponding antibodies. CtBP2 is a ZNF217-binding partner. Input from nuclear extracts is shown in the two left lanes. (PDF 99 KB)

Additional file 14: Figure S8: Overall Survival in ER^-^ and Basal breast cancer patient cohorts. A) Overall survival of basal subtype patients (n = 30) based on ZNF217 expression from primary tumors for low (n = 19) versus high (n = 11) ZNF217 expression (P = 0.01; Logrank). B). Overall survival in ER^-^ patients based on ZNF217 expression for low ZNF217 (n = 24) versus high ZNF217 (n = 17) (P = 0.005; Logrank). Data was sorted first by ZNF217 expression using the median cutoff. Then the data was sorted to identify the ZNF217 high patients and the ZNF217 low patients who were ER^-^ or basal patients. Expression Data from Chin *et al*., [36]. (PDF 27 KB)

Additional file 15: Supplementary Methods: Primers used to validate TF binding sites and Taqman Assays for RNA-seq confirmations. (XLSX 13 KB)

## Abbreviations

ZNF217: zinc finger protein #217
ER/ERα: Estrogen receptor alpha
GATA3: GATA binding protein 3
FOXA1: forkhead box A1
H3K27ac: Histone H3 acetylated on Lysine 27
H3K4me1: Histone H3 monomethylated on Lysine 4
LSD1: Histone demethylase
HDAC: Histone deacetylase
Jarid1b/Plu-1: Lysine (K)-specific demethylase
G9a: Euchromatic histone-lysine N-methyltransferase
EZH2: Enhancer of zeste homolog 2
siRNA: Small interfering RNA
ChIP-qPCR: Chromatin immunoprecipiation-quantitative PCR
MSigDB: Molecular signature database
GREAT: Genomic Regions of Enrichment Annotation Tool
TFs: Transcription factors.
