# Supplementary material for: Global analysis of ZNF217 chromatin occupancy in the breast cancer cell genome reveals an association with ERalpha
Source: BMC Genomics. 2014 Jun 24;15(1):520. doi: 10.1186/1471-2164-15-520 (PMC4082627; doi:10.1186/1471-2164-15-520)
Supplement: Supplementary file 3 — Additional file 3: Figure S1: ChIP-qPCR confirming TF binding sites identified by ChIP-seq. (A) Panel of ZNF217-bound distal sites confirmed by ChIP-qPCR. (B) Relative TF ChIP enrichment at the ERα target genes ERBB3, TFF1, and LRIG1. ChIP assays were performed on two independent biological replicates using the following antibodies ZNF217, ERα and FOXA1. IgG was used as a control. Relative DNA enrichment was calculated relative to DNA input and using the non-target ZNF10 locus as a negative control. (PDF 337 KB) [file 12864_2014_6197_MOESM3_ESM.pdf]

A

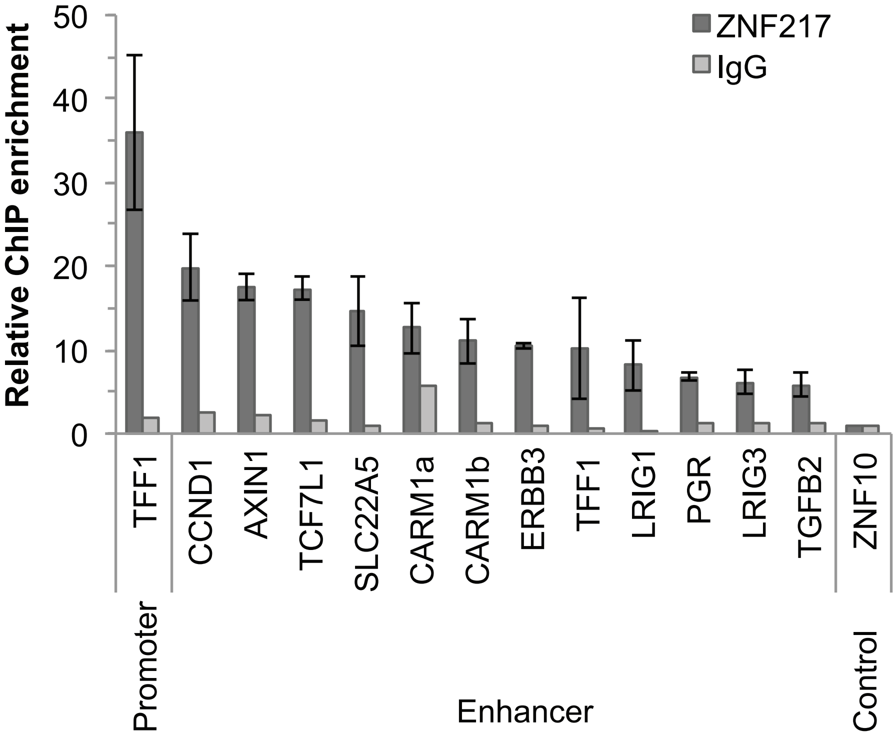

B

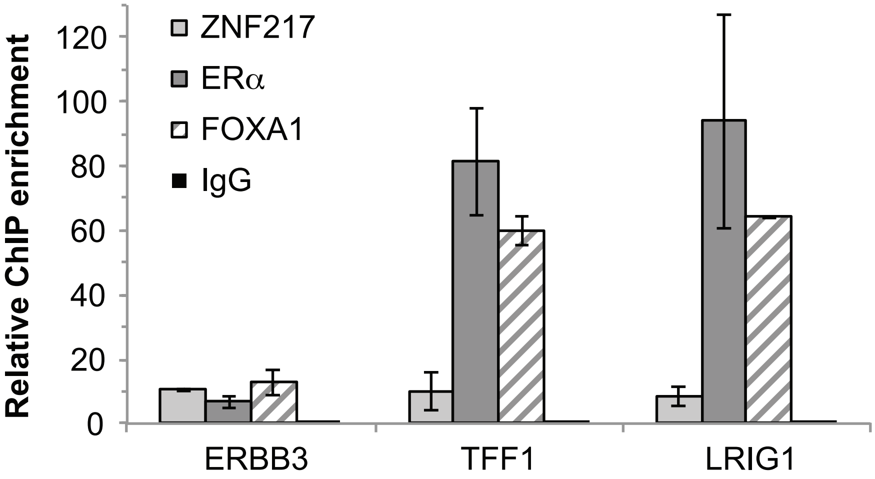

Supplemental Figure1. ChIP-qPCR confirming TF binding sites identified by ChIP-seq. **(A)** Panel of ZNF217-bound distal sites confirmed by ChIP-qPCR. **(B)** Relative TF ChIP enrichment at the ERα target genes *ERBB3*, *TFF1*, and *LRIG1*. ChIP assays were performed on two independent biological replicates using the following antibodies ZNF217, ERα and FOXA1. IgG was used as a control. Relative DNA enrichment was calculated relative to DNA input and using the non-target ZNF10 locus as a negative control.
