# Supplementary material for: Global analysis of ZNF217 chromatin occupancy in the breast cancer cell genome reveals an association with ERalpha
Source: BMC Genomics. 2014 Jun 24;15(1):520. doi: 10.1186/1471-2164-15-520 (PMC4082627; doi:10.1186/1471-2164-15-520)
Supplement: Supplementary file 4 — Additional file 4: Figure S2: Location Analysis of ZNF217 epigenomic clusters. Location analysis of the cluster I region and cluster II region from Figure 1B. The fraction of the ZNF217 binding sites found in cluster I (top chart) or cluster II (below chart) relative to Refseq genes is shown. (PDF 365 KB) [file 12864_2014_6197_MOESM4_ESM.pdf]

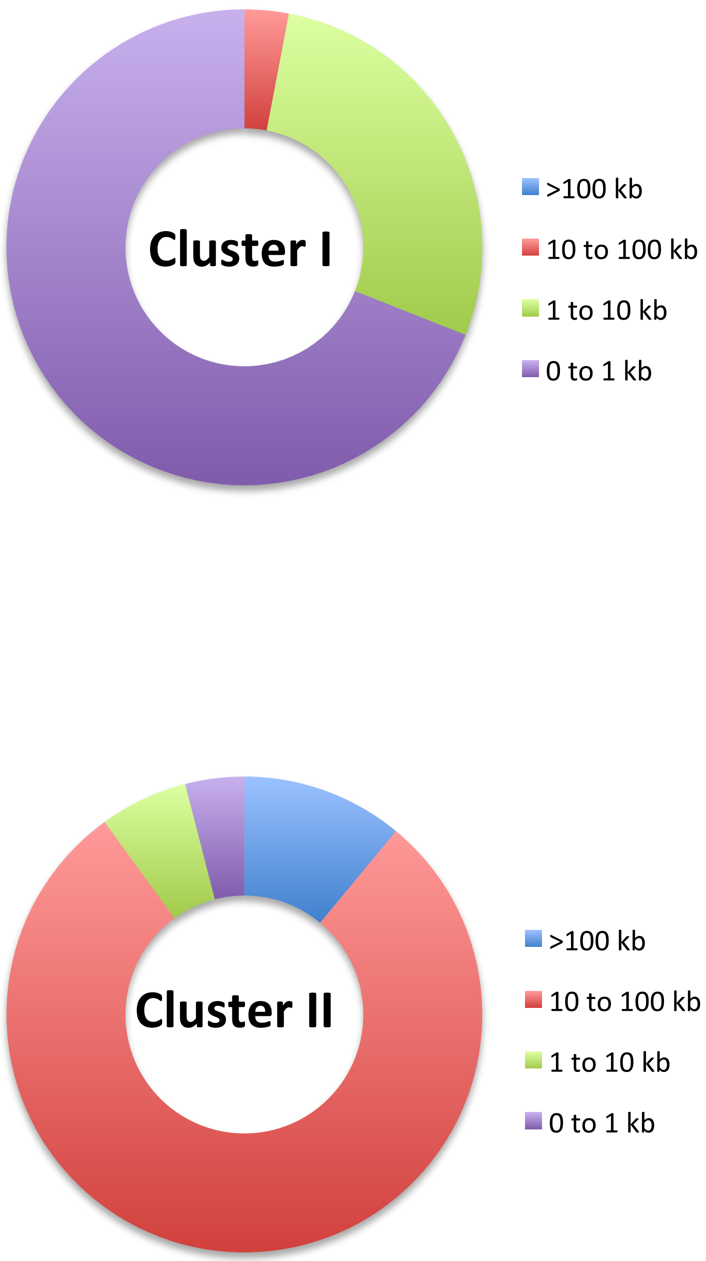

Supplmental Figure 2: Location Analysis of ZNF217 epigenomic clusters
Location analysis of the cluster I and II regions from **Figure 1B**. The fraction of the ZNF217 binding sites found in cluster I (top chart) or cluster II (below chart) relative to Refseq genes is shown.
