# Supplementary material for: Global analysis of ZNF217 chromatin occupancy in the breast cancer cell genome reveals an association with ERalpha
Source: BMC Genomics. 2014 Jun 24;15(1):520. doi: 10.1186/1471-2164-15-520 (PMC4082627; doi:10.1186/1471-2164-15-520)
Supplement: Supplementary file 6 — Additional file 6: Figure S3: de novo ZNF217 motif. Identification of de novo motif for ZNF217 binding sites in MCF7 cells. HOMER de novo motif analysis using the central most 100 base pairs of all 18,965 ChIP-seq binding regions reveal the indicated motif (shown in both forward and reverse complement). (PDF 389 KB) [file 12864_2014_6197_MOESM6_ESM.pdf]

Forward

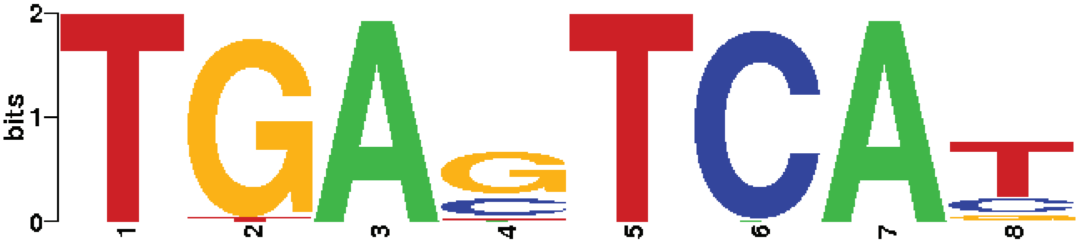

Reverse complement

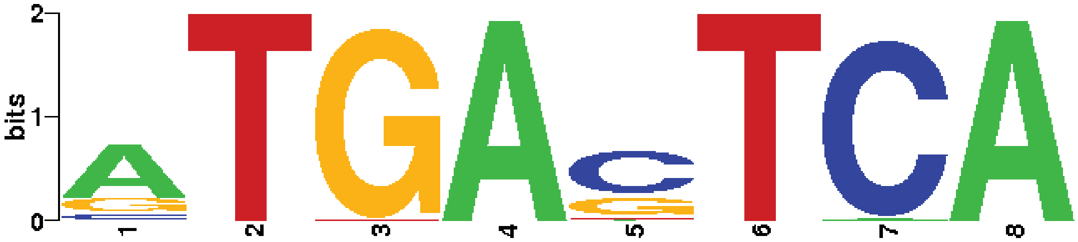

p-value:	1e-1278
Number of Target Sequences with motif	4083
Percentage of Target Sequences with motif	21.53%

Supplemental Figure 3: *de novo* ZNF217 motif
Identification of de novo motif for ZNF217 binding sites in MCF7 cells. HOMER de novo motif analysis using the central most 100 base pairs of all 18,965 ChIP-seq binding regions reveal the indicated motif (shown in both forward and reverse complement).
