# Supplementary material for: Global analysis of ZNF217 chromatin occupancy in the breast cancer cell genome reveals an association with ERalpha
Source: BMC Genomics. 2014 Jun 24;15(1):520. doi: 10.1186/1471-2164-15-520 (PMC4082627; doi:10.1186/1471-2164-15-520)
Supplement: Supplementary file 7 — Additional file 7: Figure S4: Venn diagram for ZNF217 and ER binding overlap. Overlap analysis of ZNF217 and ERα ChIP-seq binding sites in MCF7 cells. The Venn diagram illustrates the total number of genomic regions shared between these two factors (overlapping by at least 1 base pair). The total number of identified sites is indicated below each factor’s name. (PDF 355 KB) [file 12864_2014_6197_MOESM7_ESM.pdf]

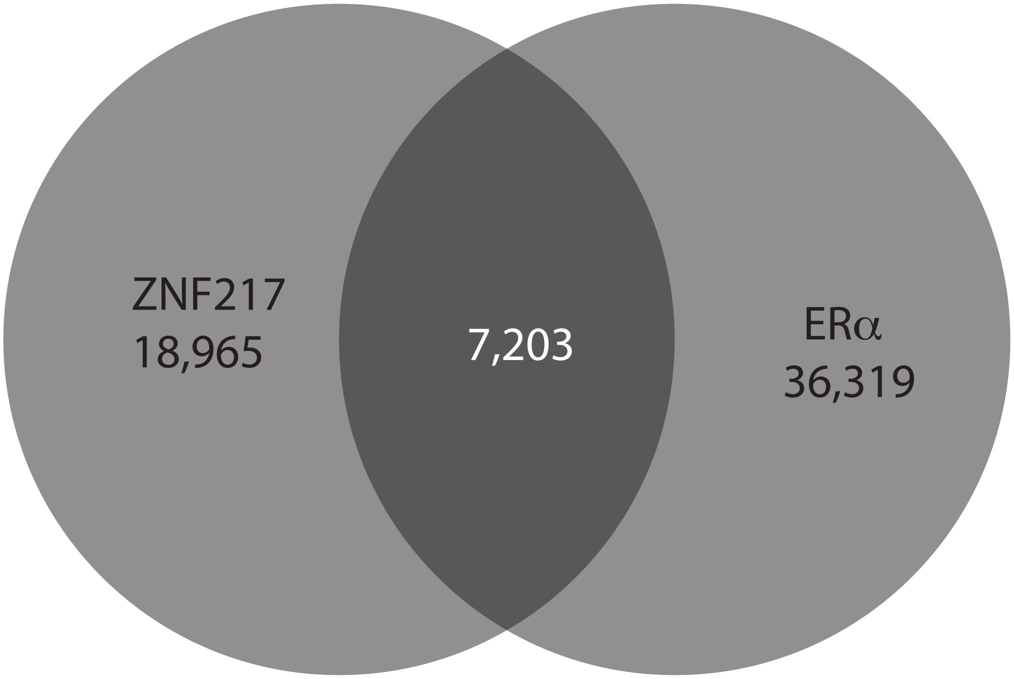

Supplemental Figure 4: Venn diagram for ZNF217 and ER binding overlap.
Overlap analysis of ZNF217 and ERα ChIP-seq binding sites in MCF7 cells. The Venn diagram illustrates the total number of genomic regions shared between these two factors (overlapping by at least 1 base pair). The total number of identified sites is indicated below each factor's name.
