# Supplementary material for: Global analysis of ZNF217 chromatin occupancy in the breast cancer cell genome reveals an association with ERalpha
Source: BMC Genomics. 2014 Jun 24;15(1):520. doi: 10.1186/1471-2164-15-520 (PMC4082627; doi:10.1186/1471-2164-15-520)
Supplement: Supplementary file 9 — Additional file 9: Figure S5: Validation of ZNF217 knockdown for RNA-sequencing. MCF7 cells were reverse transfected with scrambled or ZNF217 siRNA for 48 h. Cells were collected from each well and split into two samples for RNA isolation and protein lysate. (A) Protein lysates from triplicate samples were immunoblotted for ZNF217 or actin as a loading control. Chemoluminescence was analyzed on an Alpha-Innotech Imaging documentation system. (B) RNA samples were converted to cDNA and quantitative RT-PCR performed using ABI expression assay Taqman probes for ZNF217 and GAPDH. Each sample was assayed in triplicate and the cT values were normalized to GAPDH. Average relative transcript level was graphed using BioRad CFX software. Columns: transcript levels, error bars: standard error of the mean. (PDF 651 KB) [file 12864_2014_6197_MOESM9_ESM.pdf]

A

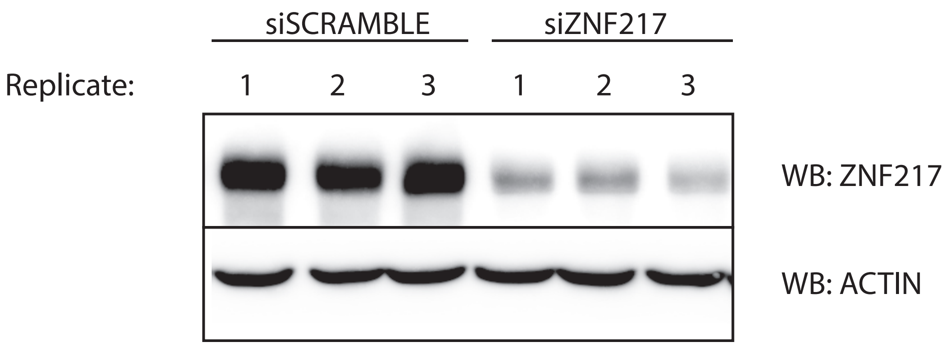

B

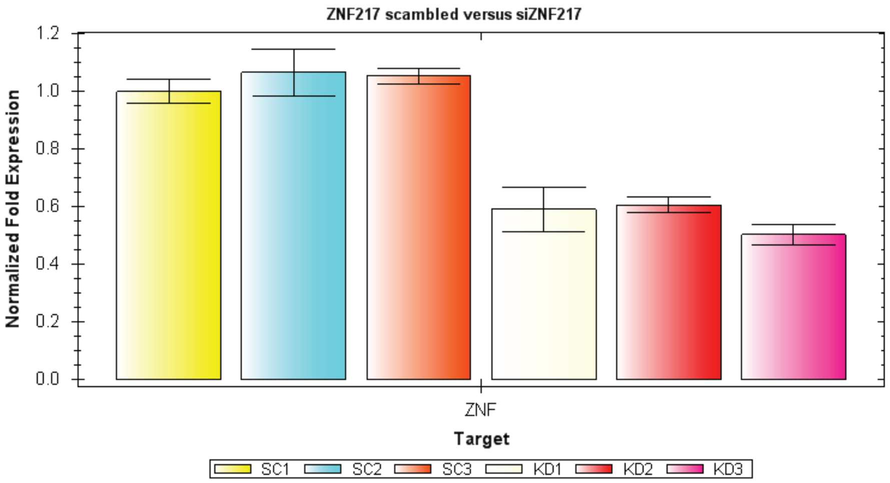

SC = siScramble
KD = siZNF217

Supplenmental Figure 5: Validation of ZNF217 knockdown for RNA-sequencing
MCF7 cells were reverse transfected with scrambled or ZNF217 siRNA for 48 h. Cells were collected from each well and split into two samples for RNA isolation and protein lysate. (**A**) Protein lysates from triplicate samples were immunoblotted for ZNF217 or actin as a loading control. Chemoluminescence was analyzed on an Alpha-Innotech Imaging documentation system. (**B**) RNA samples were converted to cDNA and quantitative RT-PCR performed using ABI expression assay Taqman probes for *ZNF217* and *GAPDH*. Each sample was assayed in triplicate and the cT values were normalized to GAPDH. Average relative transcript level was graphed using BioRad CFX software. Columns: transcript levels, error bars: standard error of the mean.
