# Supplementary material for: Global analysis of ZNF217 chromatin occupancy in the breast cancer cell genome reveals an association with ERalpha
Source: BMC Genomics. 2014 Jun 24;15(1):520. doi: 10.1186/1471-2164-15-520 (PMC4082627; doi:10.1186/1471-2164-15-520)
Supplement: Supplementary file 11 — Additional file 11: Figure S6: Integrated Genome Browser snapshots of 4 genes bound by ZNF217, ERα, FOXA1 and GATA3. Co-bound regions are shown for LRIG1, AXIN1, SLC22A5, and IGFBP4. (PDF 489 KB) [file 12864_2014_6197_MOESM11_ESM.pdf]

Frietze, Supplemental Figure 6

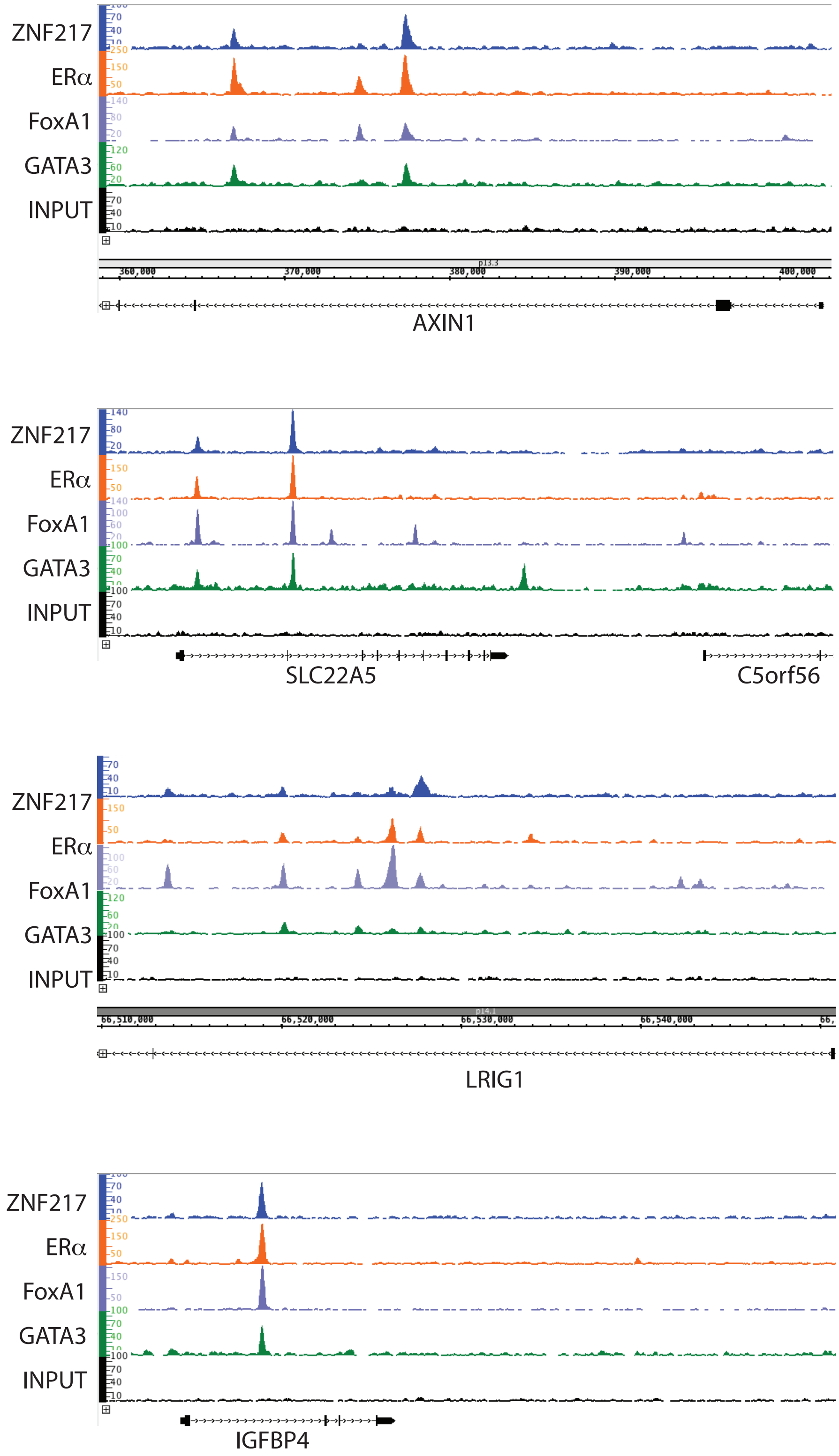


**Supplemental Figure 6:** Integrated Genome Browser snapshots of 4 genes bound by ZNF217, ERα, FOXA1 and GATA3. Co-bound regions are shown for *LRIG1*, *AXIN1*, *SLC22A5*, and *IGFBP4*.
