# Supplementary material for: Global analysis of ZNF217 chromatin occupancy in the breast cancer cell genome reveals an association with ERalpha
Source: BMC Genomics. 2014 Jun 24;15(1):520. doi: 10.1186/1471-2164-15-520 (PMC4082627; doi:10.1186/1471-2164-15-520)
Supplement: Supplementary file 13 — Additional file 13: Figure S7: ERα co-immunoprecipitation with ZNF217 and CtBP2. Co-immunoprecipitation of endogenous ZNF217, ERα and CtBP2 from MCF7 nuclear extracts. Immunoprecipitation experiments with ERα antibody to co-IP ZNF217 were blotted with corresponding antibodies. CtBP2 is a ZNF217-binding partner. Input from nuclear extracts is shown in the two left lanes. (PDF 99 KB) [file 12864_2014_6197_MOESM13_ESM.pdf]

Frietze, Supplemental Figure 7

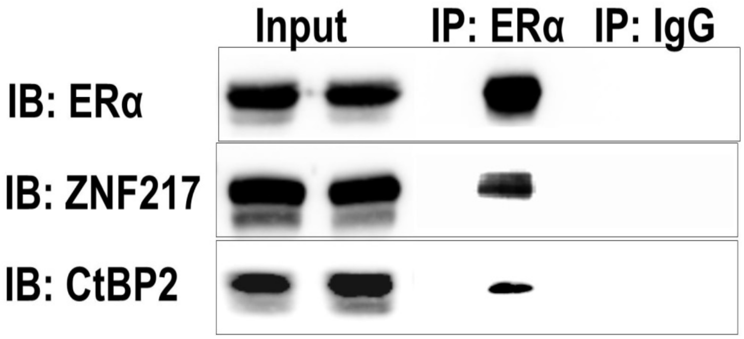

Suplemental Figure 7. ER co-immunoprecipitation with ZNF217 and CtBP2.
Co-immunoprecipitation of endogenous ZNF217, ERα and CtBP2 from MCF7 nuclear extracts. Immunoprecipitation experiments with ERα antibody to co-IP ZNF217 were blotted with Corresponding antibodies. CtBP2 is a ZNF217-binding partner. Input from nuclear extracts is shown in the two left lanes.
