# Supplementary material for: Global analysis of ZNF217 chromatin occupancy in the breast cancer cell genome reveals an association with ERalpha
Source: BMC Genomics. 2014 Jun 24;15(1):520. doi: 10.1186/1471-2164-15-520 (PMC4082627; doi:10.1186/1471-2164-15-520)
Supplement: Supplementary file 14 — Additional file 14: Figure S8: Overall Survival in ER- and Basal breast cancer patient cohorts. A) Overall survival of basal subtype patients (n = 30) based on ZNF217 expression from primary tumors for low (n = 19) versus high (n = 11) ZNF217 expression (P = 0.01; Logrank). B). Overall survival in ER- patients based on ZNF217 expression for low ZNF217 (n = 24) versus high ZNF217 (n = 17) (P = 0.005; Logrank). Data was sorted first by ZNF217 expression using the median cutoff. Then the data was sorted to identify the ZNF217 high patients and the ZNF217 low patients who were ER- or basal patients. Expression Data from Chin et al., [36]. (PDF 27 KB) [file 12864_2014_6197_MOESM14_ESM.pdf]

Frietze, Supplemental Figure 8

A

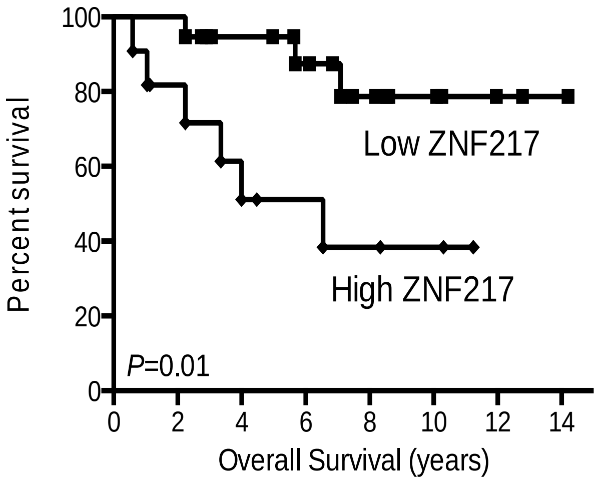


B

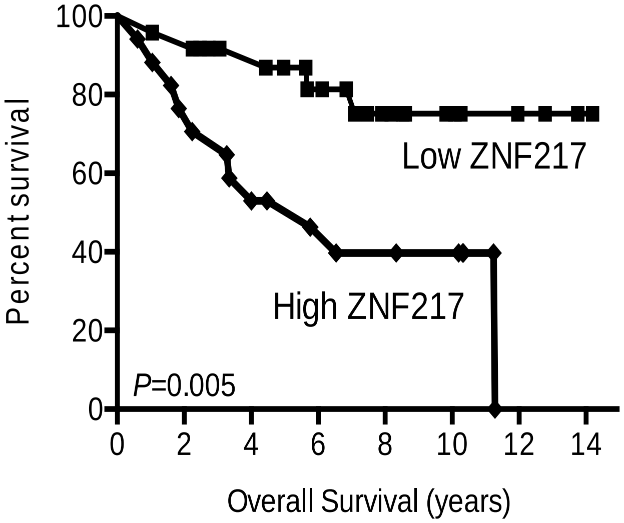


**Supplemental Figure 8. Basal and ER- patient cohorts**
**A)** Overall survival of basal subtype patients (n=30) based on ZNF217 expression from primary tumors for low (n=19) versus high (n=11) ZNF217 expression (P=0.01; Logrank). **B)** Overall survival in ER- patients based on ZNF217 expression for low ZNF217 (n=24) versus high ZNF217 (n=17) (P=0.005; Logrank). Data was sorted first by ZNF217 expression using the median cutoff. Then the data was sorted to identify the ZNF217 high patients and the ZNF217 low patients who were ER- or basal patients. Datasets from Chin et al., 2006.
